# Cardiogenic shock as a complication of acute mitral valve regurgitation following posteromedial papillary muscle infarction in the absence of coronary artery disease

**DOI:** 10.1186/1749-8090-3-61

**Published:** 2008-11-04

**Authors:** Federico Bizzarri, Consalvo Mattia, Massimo Ricci, Flaminia Coluzzi, Vincenzo Petrozza, Giacomo Frati, Giuseppe Pugliese, Luigi Muzzi

**Affiliations:** 1 Cardiac Surgery Unit-Polo Pontino, Heart and Great Vessels Department, University of Roma "Sapienza", Via F. Faggiana 34, Latina-Italy; 2 Anaesthesiology and Intensive Care Unit, Rome University "Sapienza"-Polo Pontino, Via F. Faggiana 34, Latina-Italy; 3 Pathology Unit, Rome University "Sapienza", Via F. Faggiana 34, Latina-Italy

## Abstract

A 48 year old man was transferred to our department with cardiogenic shock, pyrexia, a high white cell count and significant serum troponin T level. Clinical evaluation revealed severe mitral regurgitation secondary to a flail of both mitral valve leaflets. An emergency cardiac catheterisation did not reveal any significant coronary artery disease. Left ventricular angiogram and echocardiography demonstrated a good left ventricular function and massive mitral regurgitation. Blood cultures were negative for aerobics, anaerobics and fungi. The patient underwent emergency mitral valve replacement with a mechanical valve. Intraoperatively, the posteromedial papillary muscle was found to be ruptured. Histology of the papillary muscle revealed myocardial necrosis with no signs of infection. Cultures obtained from a mitral valve specimen were negative. The patient's recovery was uneventful and he was discharged on the 6^th ^postoperative day.

## Case report

0.4%–5% of patients with acute myocardial infarction die from sudden and severe mitral regurgitation due to a complete rupture of a papillary muscle [1]. Most commonly, the posteromedial papillary muscle is involved, but in 20% of cases, the anterolateral papillary muscle is involved. The muscle rupture may represent a rare mechanical complication of a blunt chest trauma, or may be due to sepsis [2].

Spontaneous rupture of the papillary muscle, secondary to isolated papillary muscle infarction, in the absence of coronary artery disease, is incommon or rare.

We describe a case of a spontaneous acute papillary muscle rupture in the absence of a history of ischemic cardiopathy or blunt chest trauma or sepsis.

A 48 year old man was transferred to the intensive care unit in June 2008 with signs and symptoms of cardiogenic shock after extreme exercise. He was a farmer and had been lifting a bale of corn. He collapsed immediately after exercise. Past medical history was positive for heavy smoking, severe arterial hypertension and hypercholesterolemia. There was no previous history of chest pain, heart murmurs or drug abuses. Up until the even the patient had appeared to be in good health and had not been on medication. There was no history of trauma to the chest.

Physical examination revealed congestive heart failure and a loud pan-systolic apical murmur, radiating posteriorly. The ECG showed sinus tachycardia. Chest X-ray showed pulmonary venous congestion and was suspicion of an infiltrate in the right lower lobe which could be consistent with the patient's pyrexia. An urgent echocardiogram showed normal systolic function, severe left ventricular hypertrophy (14 mm septum wall), an increased end-diastolic diameter of the left ventricle (58 mm) and posterior and anterior mitral valve prolapse with severe mitral valve regurgitation. Coronary angiography and a left ventricular angiogram showed normal coronary arteries and a good left ventricular function with massive mitral regurgitation. The patient was pyrexyal with a temperature of 38.6°. Laboratory tests showed an elevated white cell count (18 × 10^3^/mm^3^) and elevated troponin T level (1.5 ng/ml).

Cultures (blood, sputum, urine) were taken and broad-spectrum antibiotics were started.

The patient underwent emergency surgery. At operation the head of the posteromedial muscle was found to be ruptured, leading to a complete flail of the posterior and anterior mitral leaflets. Histology of papillary muscle specimens revealed lesions comparable to that of an acute myocardial infarction. Valve replacement was carried out with a mechanical bileaflet valve. Weaning from cardiopulmonary bypass was uncomplicated and the patient's recovery was uneventful. Blood cultures for anaerobics, aerobics and fungi were negative, as were serological tests done for other inflammatory conditions, such as rheumatoid arthritis.

## Discussion

Isolated rupture of the papillary muscle, which is extremely atypical in the absence of coronary abnormalities is usually secondary to a myocardial infarct or rarely to a blunt chest trauma. In the literature, a case of rupture of the papillary muscle as a consequences of sepsis due to clostridium in a patient with SLE (systemic lupus erythematosus) and antiphospholipid syndrome [3] has been described. Because of the low incidence of the disease the exact pathogenesis is unclear. In cases of left ventricular hypertrophy, stress can lead to a relatively more severe ischemia of the papillary muscle in view of a relatively lower coronary circulation in relation to muscle size and cardiac fibrosis [4].

It is possible that a temporary occlusion of the coronary artery selectively irrigates the papillary muscle with a subsequent ricanalization of the artery, but the occlusion can last long enough to condition an infarct of the myocardium, which can be identified by clinical findings and by elevated enzyme levels. This could form an explanation in the case of our patient.

The posteromedial papillary muscle is affected more often than the anterolateral papillary muscle because the latter receives blood both from the diagonal branches of the descending left anterior artery and the marginal branches of the curve. The posteromedial muscle owes its blood supply only to the posterior interventricular artery. The infarct is primarily subendocardial in approximately half of the cases of muscular rupture, and trans-mural in the other half. It is usually a small infarct. In some cases rupture of the papillary muscle is associated in its development both with a defect of the interventricular septum and the rupture of the free wall of the left ventricle.

Surgical mortality is high and, according to case studies, fluctuates between 19% and 85%. Mortality is influenced by the type of surgical technique, both in repair and replacement procedures. Surgical technique influences long-term survival, as highlighted by the studies carried out by Gillinov et al [5].

## Competing interests

The authors declare that they have no competing interests.

## Authors' contributions

FB, GF, GP, LM and MR are members of surgical team. CM and FC were the anesthesist involved in theatre and in intensive care unit. VP was the pathologist. All authors have read and approved the final manuscript.

## Consent

Written informed consent was obtained from the patient for publication of this case report. A copy of the written consent is available for review by the Editor-in-Chief of this journal.
